# Presidential address: Ahmedabad, 23^rd^ January 2010

**DOI:** 10.4103/0971-3026.63039

**Published:** 2010-05

**Authors:** Kishor Taori

**Affiliations:** President, Indian Radiological and Imaging Association, 2010, Department of Radiodiagnosis, Govt Medical College and Hospital, Nagpur 440 003, Maharashtra, India. kishortaori@yahoo.co.in


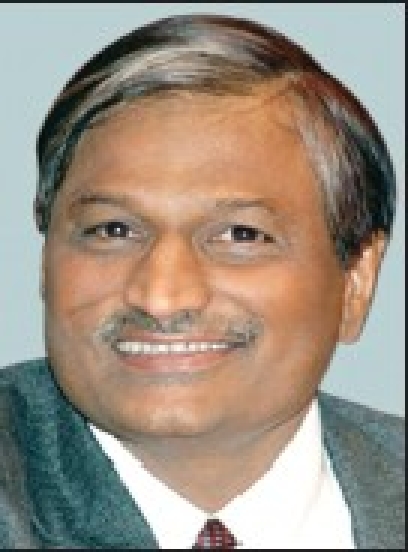


Honorable chief guest Dr. Jaynarayan Vyas, Health Minister, Gujarat State, my learned predecessor, outgoing President Dr. Prabhakar Reddy, dignitaries on the dias and off the dias, organizing chairman Dr. Harshad Shah, distinguished guests from India and abroad, IRIA colleagues, my Vidarbha colleagues, students, representatives from the health care industry, media and ladies and gentlemen. It is a matter of great honor and pride to address this august gathering as the president of IRIA, which is one of the greatest radiology organizations of the world.

I am indeed happy today to be installed at Ahmedabad, the pious and holy land of Mahatma Gandhi, with its rich cultural values and traditions. This prosperous state of Gujarat is in a position today to inspire the other states in our country in many spheres and walks of life.

Our esteemed association took birth in the year 1931 in Kolkata and now entering its 79^th^ year; it is becoming more and more vibrant with age. With an increase in our membership to more than 8000, the association is becoming younger in terms of mean age.

It is for all of us to appreciate the continuous efforts, which have been put in by my predecessors and their teams to bring this association to this level. We are proud to have our own building at New Delhi, our academic wing ICRI, and our journal, which has recently been indexed with PubMed. I congratulate the excellent work done by all the office bearers.

## Relationship Toward the Statutory Organization

As one of the biggest organizations in modern medicine, IRIA should have a good liaison with local corporations, respective state medical councils, and the Medical Council of India as well as other government organizations.

Modern Radiology is a golden leaf that is added as a chapter to text-books belonging to various other subjects such as Medicine, Surgery, Orthopedics, etc. at both undergraduate and postgraduate levels. None of the theory and practical examinations can do without the involvement of radiological imaging. The scope and interpretation of radiology at the postgraduate level is still higher and it is part and parcel of all other curricula. Such scope and its essential role in precise diagnosis can no longer be neglected. The planners at both the University and the MCI levels will have to be concerned and realistic so as to bring in radiology teaching at the undergraduate level in some or the other form and also in all the non-radiology postgraduate courses.

It will be our endeavor to participate in the shaping of the modern curriculum in the field of radiology at the postgraduate level. We need to prescribe standard imaging modalities, which should be installed in all teaching departments where the MD and DMRD courses are in operation.

## PC-PNDT Act

The provisions of the PC-PNDT Act for “save the girl child” are good on paper, but have proven illogical on many occasions, causing harassment to the radiologist, without having any desired results in the direction contemplated.

I will strive hard for the strict implementation of the PC-PNDT Act without causing any harassment to our honest doctors. IRIA will work in close liaison with the IMA for proper education of society to change its mindset that a boy or a girl give the same value, pleasure, and demand equality, in terms of parenting.

It will be our endeavor to convince the authorities to agree to differential punishment for minor and major breaches of the Act. Presently, a simple miss of a signature on any form can result in seizure of a machine. The participation and relevant inputs are earnestly solicited from all the members of IRIA in understanding the PC-PNDT Act and smooth implementation of the same.

## Radiology Practice

My dear members of IRIA, we are all fortunate to be radiologists. Radiology is at its zenith and no patient in the Government or private practice can remain untouched from a radiologist's interpretation. With the armamentarium at our disposal, we have almost become indispensable and have a concrete place in modern management and health care.

The expectations of society at large are increasing. Now the equation that “necessity is the mother of invention” has changed to “invention is the mother of necessity.” The other side of the coin is “the cost.” The planners, the Government, representatives of the trade, and IRIA will have to apply their mind to bring the fruits of modern radiology to the doorstep of the masses at an affordable cost. It is important that some norms for fair practice in radiology are also established by IRIA and followed by our members to enhance the image of our community.

The National Rural Health mission has given tele-radiology hubs to all the regional institutes, which are in turn connected to the district hospitals. Further, the application of tele-radiology may descend to rural hospitals with the installation of digital equipments making the job of image transfer easier. In Maharashtra, we have already requested the State Government to establish a telemedicine hub at every big IMA branch, wherein the services of our IRIA members could be utilized for image interpretation.

## Elevating the Next Generation

More than 50% of radiologists in India are below the age of 45. With many young radiologists including female radiologists, I can sense a further rise in dynamism and youthful creative ideas in our profession. How will the radiologists of the coming generation look at us? Will they really marvel at our achievements today? Will they really admire us, applaud us, be grateful for the work we are doing today, which will help shape their futures by providing enough direction? Would they be reading our record of nice work?

We will be judged by our future generation not for what has been done but for what has been left undone. So let us rise from this center stage and utilize this chance to its maximum, to make a mark in the future annals of IRIA. I do not know whether the promise will be fulfilled. Maybe the goal will recede and pull us along or it may be realized and even surpassed.

With the recent advances in radiodiagnosis like PET scan, HIFU, Interventional Radiology, it is also expected from young radiologists that they should take-up different subspecialties in radiology but at the same time take precautions not to develop tubular vision.

## Educational Conferences

It is necessary for every one of us to be updated about the widening horizons of radiology. Every coming day has new challenges for all of us. There is a continuous organization of CMEs in different parts of the country with wide participation. All the state chapters and subchapters are doing their best in updating consultants and students as well. However more focused, well-structured CMEs with precision are necessary to make them worthy of international standards.

It will be our request to the MCI to sanction accreditation for national conferences and other CME programs with stipulated credit hours. As the world is now a global village with effective communication, international liaison is easy and exchange of knowledge and ideas a realistic possibility, today.

Our Academic wing, the Indian College of Radiology has been doing excellent work under Dr. Bharat Parekh and Dr. Deepak Patkar. I wish that all the academic programs and CMEs be handled by the College and more and more people should participate and become members of ICRI.

There is a regular funding program from the University Grants Commission for sponsoring teachers for reading papers in standard national conferences. MCI also funds the teachers to read their research papers in international conferences as well as for organizing CME programs. IRIA is also coming forward in a similar way to promote participation in international conferences by our members.

In the past, we have been able to host international conferences and also participate in overseas conferences. We have good coordination with a few developed countries for provision of short-term fellowships at their ends, for young radiologists from our country. I sincerely request representatives, Presidents, secretaries of the Radiology Associations of different countries, to help the progress of radiology in India, by offering more numbers of fellowships to our young radiologists. Your gesture will go a long way in raising global standards.

We have experienced a taste of the lavish and unforgettable hospitality and the great show put up by the organizers of the 63^rd^ Annual Congress of IRIA. My heartiest thanks to the Chairman, Dr. Harshad Shah, for his untiring and inspiring efforts. I appreciate the efforts of Dr. Dinesh patel, Dr. Y.T Patel, Dr. Hemant Patel, and all the commanders of the organizing committee for their time, energy, money, and for acting in tandem for the cause of our organization.

I express my gratitude to the captains of the trade for giving support and cooperation towards the progress of our association.

This year IRIA is hosting the SAARC conference at Nagpur on the 7^th^ and 8^th^ of August 2010. Friends, I extend a warm invitation to all for your valuable contribution and participation.

I am grateful to everyone who has helped me reach this coveted post. I am not in a position to single out anyone, as there are many silent workers extending their helping hands at all the times. Friends, I bow down with utmost humility to thank you. I will strive hard to create a deserving place in your heart for your love and affection.

Please continue to support me in the areas in which you have a better hold. Every one of us has potential, means, and objectives as well. However these are needed to be exploited for the cause of the IRIA too. Please do that and participate. Our unequivocal flow of thought and multidirectional approach will make IRIA strong.

I am obliged by my family members for their perfect sense of understanding and their support, which has helped me stand before you. My wife Dr. Bharti and children Abhijeet and Abhishek deserve special appreciation from me. I am fortunate to be lovingly blessed today by my father Dr. Badridasji Taori and I remember my dear mother, late Kamaladevi Taori, who always encouraged me throughout her life.

I assure you that I will work with a positive mindset for the cause of radiology in general and IRIA in particular.

Once again, I extend my best wishes to you and your family for a bright and healthy 2010.

Thank you very much, ladies and gentlemen.

Long Live IRIA

